# Validation of a clinical rotation evaluation for physician assistant students

**DOI:** 10.1186/s12909-018-1242-y

**Published:** 2018-06-04

**Authors:** Ryan A. Meverden, Jason H. Szostek, Saswati Mahapatra, Cathy D. Schleck, Jayawant N. Mandrekar, Thomas J. Beckman, Christopher M. Wittich

**Affiliations:** 1 Mayo Clinic Gonda Vascular Center, Mayo Clinic, Rochester, MN USA; 2 Division of General Internal Medicine, Mayo Clinic, 200 First St. SW, Rochester, MN 55905 USA; 3 Division of Biomedical Statistics and Informatics, Mayo Clinic, Rochester, MN USA

**Keywords:** Assessment, Clinical rotation, Physician assistant education, Validation study

## Abstract

**Background:**

We conducted a prospective validation study to develop a physician assistant (PA) clinical rotation evaluation (PACRE) instrument. The specific aims of this study were to 1) develop a tool to evaluate PA clinical rotations, and 2) explore associations between validated rotation evaluation scores and characteristics of the students and rotations.

**Methods:**

The PACRE was administered to rotating PA students at our institution in 2016. Factor analysis, internal consistency reliability, and associations between PACRE scores and student or rotation characteristics were determined.

**Results:**

Of 206 PACRE instruments sent, 124 were returned (60.2% response). Factor analysis supported a unidimensional model with a mean (SD) score of 4.31 (0.57) on a 5-point scale. Internal consistency reliability was excellent (Cronbach α=0.95). PACRE scores were associated with students’ gender (*P* = .01) and rotation specialty (*P* = .006) and correlated with students’ perception of being prepared (*r* = 0.32; *P* < .001) and value of the rotation (*r* = 0.57; *P* < .001).

**Conclusions:**

This is the first validated instrument to evaluate PA rotation experiences. Application of the PACRE questionnaire could inform rotation directors about ways to improve clinical experiences. The findings of this study suggest that PA students must be adequately prepared to have a successful experience on their rotations. PA programs should consider offering transition courses like those offered in many medical schools to prepare their students for clinical experiences. Future research should explore whether additional rotation characteristics and educational outcomes are associated with PACRE scores.

## Background

Physician assistants (PAs) are vital to all aspects of health care delivery. The number of PA training programs continues to increase to meet the demand for access to health care [1–3]. In the past decade, the number of accredited PA programs and applicants to these programs has increased dramatically [4, 5]. An integral part of these programs are clinical rotations, which are limited by competition and shortages [6–9]. The educational quality of these clinical rotations can vary [10]. Maintaining standards in clinical rotations, including validated assessment of performance, is a prerequisite to ensuring a high-quality PA workforce.

Data are sparse regarding the evaluation of training programs and individual clinical rotations in other educational settings. In medical school, students have evaluated clerkships using electronic Likert-scaled checklists [11]. In graduate medical education, residents have used validated questionnaires to evaluate their programs with respect to rotation workload, faculty/learning environment, and stress [12–15]. Other studies have examined resident assessments of programs and rotations in surgery [16], internal medicine [17, 18], and anesthesiology [19]. Although PAs are trained in the medical model, differences in clinical rotation length, content, supervision, and logistics make the use of existing clinical rotation evaluations less relevant to PA training settings. We are unaware of prior research on validated measures of PA clinical rotations.

To fill this gap, we conducted a prospective validation study to develop a PA clinical rotation evaluation (PACRE) instrument. The specific aims of this study were to 1) develop a tool to evaluate PA clinical rotations, and 2) once validated, explore associations between PACRE rotation evaluation scores and characteristics of the students and rotations. The purpose of the PACRE instrument was to determine and document a student’s perceptions of a rotation based on research on components of effective clinical teaching. We hypothesized that such an instrument would have strong internal structure validity and scores would be associated with rotation or student demographic variables.

## Methods

### Participants and clinical sites

This prospective validation study involved PA students who completed a clinical rotation in 2016 at1) the Mayo Clinic School of Health Sciences/Gundersen Medical Foundation/University of Wisconsin – La Crosse PA Program or 2) the Mayo Clinic or the Mayo Clinic Health System from other PA programs. The study was deemed exempt by the Mayo Clinic institutional review board (Identification number: 15–006040).

### PACRE instrument development

A PACRE questionnaire was developed on the basis of existing literature [12, 14, 15, 20–28]. Items were developed for each of the Stanford Faculty Development Program (SFDP) for Clinical Teaching categories: learning climate, control of session, communication of goals, promotion of understanding and retention, evaluation, feedback, and promotion of self-directed learning [24, 29]. Two additional categories—rotation logistics and a rotation global assessment—were included. After iterative revision, 2 items were selected for each of the 9 domains, for a total of 18 items in the PACRE instrument (Table 1). Responses were based on a 5-point Likert scale (1, strongly disagree, to 5, strongly agree). The final PACRE instrument was pilot tested on 5 former PA students and 5 current PA colleagues, which led to minor rewording of some items.

**Table 1:** PACRE Instrument Educational Domains, Items, Item Loadings, and Mean Scores

Educational Domain	PACRE Item	Item Loading^c^	Mean (SD) PACRE Score
Learning climate	The preceptor (s) created an environment that was conducive to learning.	0.7958	4.40 (0.75)
	The preceptor (s) was/were enthusiastic.	0.6585	4.56 (0.71)
Control of session	The preceptor (s) balanced time between patient care and teaching.	0.7751	4.35 (0.85)
	The preceptor (s) utilized my time effectively.	0.7515	4.24 (0.81)
Communication of goals	The rotation goals were stated clearly.	0.7937	4.20 (0.82)
	The rotation goals were appropriate for my educational needs.	0.7108	4.38 (0.69)
Promotion of understanding and retention	The educational content was clearly communicated.	0.7536	4.33 (0.70)
	The preceptor (s) helped to facilitate my understanding and retention of information.	0.7770	4.47 (0.65)
Evaluation	My performance was assessed by the preceptor (s).	0.8028	4.26 (0.72)
	I was evaluated on what I learned.	0.7788	4.08 (0.81)
Feedback	I received feedback on my performance.	0.8524	4.22 (0.75)
	The preceptor (s) communicated constructive assessments of my abilities.	0.8188	4.22 (0.79)
Promotion of self-directed learning^a^	I had access to educational resources.	0.2183	4.68 (0.49)
	I was encouraged to learn on my own.	0.5270	4.54 (0.55)
Rotation logistics	I was oriented to information regarding rotation logistics.	0.7360	4.16 (0.75)
	My overall experience was organized.	0.7626	4.37 (0.68)
Global assessment	I would recommend this rotation.	0.7463	4.36 (0.82)
	I would recommend this site/work area as a place of employment.	0.6587	4.41 (0.75)
Overall (16 items)^b^			4.31 (0.57)

Abbreviation: *PACRE* physician assistant clinical rotation evaluation

^a^Items from this educational domain loaded ambiguously and were excluded.

^b^Cronbach α (internal consistency reliability) was 0.95 for the 16 retained items.

^c^The eigenvalue for factor 1 was 9.63. Eigenvalues for all additional factors were < 1, which supports a one factor model for the PACRE instrument.

### Data collection and analysis

The PACRE was sent via an emailed link to each PA student at the completion of the clinical rotation. Our survey research center managed the data collection using Qualtrics software (Qualtrics LLC). A reminder email was sent 1 week after the completion of the rotation.

Demographic characteristics were collected, including gender, age, and number of previous rotations. Rotation characteristics were collected, including rotation specialty (general practice, medicine subspecialty, medical specialty, pediatrics, surgery, other), rotation type (required, elective), and length of rotation (4 or 6 weeks). These demographic and rotation characteristics were chosen based on the authors’ hypothesis for potential associations with PACRE scores and the availability of accurate date. Each student ranked the following questions on a 5-point Likert scale: 1) My program adequately prepared me for this rotation; and 2) This rotation prepared me for being a PA.

Factor analysis was completed on the PACRE instrument item scores. “To account for the clustering of multiple ratings by students completing more than 1 rotation evaluation, we generated an adjusted correlation matrix using generalized estimating equations. This adjusted correlation matrix was then used to perform factor analysis with orthogonal rotation. For a sensitivity analysis, we performed a factor analysis using an unadjusted correlation matrix and within-student combinations” [30]. Specifically, the sensitivity analysis involved conducting the factor analysis at lowest level of measurement (the student) and also higher levels of measurement (group-averaged scores), and then comparing these different levels of analysis to determine if they reveal similar or identical factor structures, which would then support reporting factor analysis of the higher, nested level of measurement. The number of factors to be retained was determined based on the eigenvalue criterion (factors with eigenvalues > 1). The final model was confirmed by reviewing the scree plot. Items with factor loadings ≥0.60 were retained. Internal consistency reliability was calculated using the Cronbach α, where α greater than 0.7 is considered acceptable [31]. Additionally, for internal structure validity determination an evaluation-to-item ratio should range from 5:1 to 10:1 [31]. The 18 items that make up the PACRE instrument would require between 90 and 180 completed instruments in order to be powered to complete the factor analysis.

Categorical variables are presented as count (percentage) and continuous variables are presented as mean (SD). Associations between PACRE instrument scores and categorical student demographic and clinical rotation characteristics were determined using the Wilcoxon rank sum test (if 2 groups) or Kruskal-Wallace test (if more than 2 groups). Pearson correlation coefficients were calculated to explore the relationship between PACRE scores and continuous participant or rotation characteristics (0.1–0.3, small correlation; 0.3–0.5, medium correlation; 0.5–1, large correlation) [32]. Given multiple comparisons, the threshold for statistical significance was set at *P*≤.01. Statistical analysis was conducted using SAS version 9.3 software (SAS Institute, Inc.).

## Results

### Participant characteristics

Of 206 surveys sent to 41 unique PA students, 124 surveys were returned (60.2% response rate) by 33 students. Of the responses, 118 surveys from 32 students contained both evaluation and demographic data, and these are the data source for this study. There were 28 students from the La Crosse program and 4 students were from other PA programs. The 32 students completed between 1 and 7 rotation evaluations. Student demographics are shown in Table 2.

**Table 2:** Associations Between PACRE Scores and Student or Clinical Rotation Characteristics

Characteristic	Value^a^ (*N* = 118)	PACRE Score^b^	*P* Value
Student characteristics			
Gender			.01^c^
Men	10 (8.5)	4.7 (0.4)	
Women	108 (91.5)	4.3 (0.6)	
Age, y	24.1 (1.8)	4.3 (0.6)	.07^d^
No. of previous rotations	5.3 (3.0)	4.3 (0.6)	.24^d^
Clinical rotation characteristics			
Specialty			.006^c^
General practice	30 (25.4)	4.6 (0.5)	
Medicine subspecialties ^e^	10 (8.5)	4.5 (0.4)	
Medical specialties ^f^	24 (20.3)	4.2 (0.6)	
Pediatrics	9 (7.6)	4.2 (0.6)	
Surgery	29 (24.6)	4.1 (0.5)	
Other ^g^	16 (13.6)	4.2 (0.6)	
Type			.85^c^
Required	84 (71.2)	4.3 (0.6)	
Elective	34 (28.8)	4.3 (0.6)	
Location			.46^c^
Tertiary center	76 (64.4)	4.3 (0.6)	
Health system	42 (35.6)	4.4 (0.6)	
Length of rotation, wk			.39^c^
4	115 (97.5)	4.3 (0.6)	
6	3 (2.5)	4.6 (0.4)	
My program adequately prepared me for this rotation ^h^	4.2 (0.8)	4.3 (0.6)	<.001^d^
This rotation prepared me for being a PA	4.4 (0.7)	4.3 (0.6)	<.001^d^

Abbreviations: *PA* physician assistant, *PACRE* physician assistant clinical rotation evaluation

^a^Values are No. of responses (%) or mean (SD).

^b^Values are mean (SD).

^c^Wilcoxon rank sum test (2 groups) or Kruskal-Wallace test (> 2 groups).

^d^Pearson correlation.

^e^Cardiology [9], Endocrinology [1].

^f^Dermatology [5], Emergency Medicine [7], Neurology [2], Psychiatry [10].

^g^Geriatrics [1], Hematology/Oncology [4], Infectious Disease [4], Interventional Radiology [1], Pain Management [2], Urology [2], Vascular Medicine [1], Women’s Health [1].

^h^Pearson correlation coefficient, 0.32.

^i^ Pearson correlation coefficient, 0.57.

### PACRE instrument validation

**Fig. 1:**
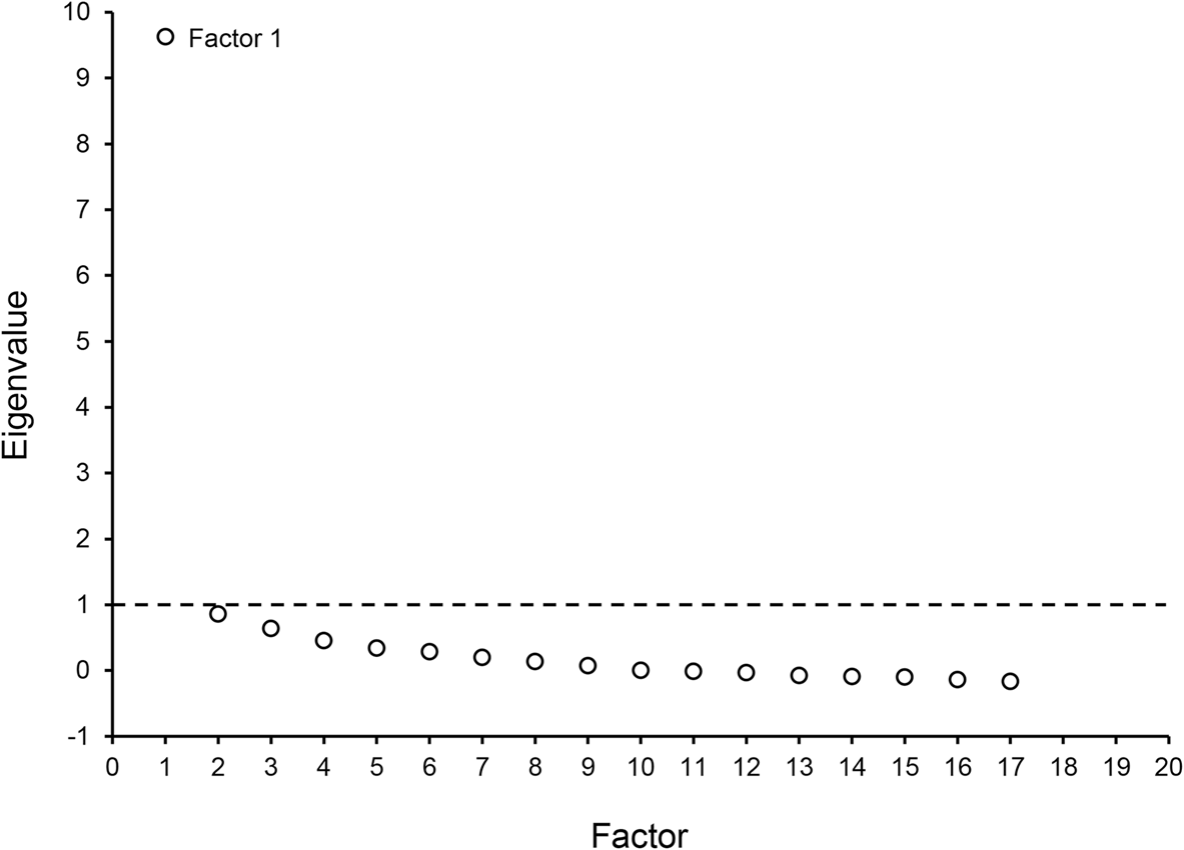
Scree plot for the one factor model in the PACRE instrument. The scree plot shows the eigenvalues for the factors and supports the decision to retain the factors with an eigenvalue > 1

Factor analysis of the PACRE instrument showed a unidimensional model for assessing PA clinical rotations (Table 1). The eigenvalue for the PACRE instrument’s one factor was 9.63. Eigenvalues for all additional factors were < 1, which supports a one factor model (Fig. 1). Item factor loadings were all higher than 0.6, except for 2 items developed for “promotion of self-directed learning.” These items were removed from the remainder of the analysis and future iterations of the PACRE instrument. The internal consistency reliability was excellent (Cronbach α=0.95). The item mean (SD) scores ranged from 4.08 (0.81) to 4.56 (0.71). The mean overall PACRE score was 4.31 (0.57).

### Associations between PACRE scores and student or rotation characteristics

PACRE scores were associated with the students’ gender. Men gave significantly higher PACRE scores than women (mean [SD], 4.7 [0.4] vs 4.3 [0.6]; *P* = .01). There were no significant associations between rotation evaluation scores and student age or PA program (tertiary center vs health system).

PACRE scores were associated with the specialty of the rotation. General practice rotations had the highest PACRE scores (4.6 [0.5]), and surgical rotations had the lowest (4.1 [0.5]; *P* = .006). There were no significant associations between rotation evaluation scores and rotation type or length.

Analysis indicated significant relationships between PACRE scores and Likert-scaled responses to 1) My program adequately prepared me for this rotation (Pearson correlation coefficient, 0.32; *P* < .001) and 2) This rotation prepared me for being a PA (Pearson correlation coefficient, 0.57; *P* < .001). These represent a medium correlation and a large correlation, respectively [32].

Regarding statistical power, for a binary variable with a prevalence of 50%, the sample size of 118 students has 80% power to detect a moderate effect size of 0.52 standard deviations or higher based on a two sample of t-test with equal variance. For continuous variables, a sample of size 118 students has 80% power to detect a correlation coefficient of 0.26 or higher between any two continuous variables, such as age versus PACRE score.

## Discussion

To our knowledge, this is the first study to report the validity of a clinical rotation assessment in PA education. The newly developed PACRE questionnaire represents a unidimensional model with strong internal consistency reliability. Student rotation evaluation scores were associated with the students’ gender and specialty of the rotation but not with whether it was required or elective. There was a positive correlation between the rotation evaluation scores and whether the student felt prepared for the rotation and whether they felt the rotation prepared them to be a PA.

This study adds to what is known about evaluations of clinical teaching and rotations. Research on assessments of clinical teaching in other educational settings has been previously published. Factorial validation of the SFDP core components among medical students resulted in the SFDP26, a questionnaire that consists of 25 items organized around these competencies and 1 item for overall effectiveness [24]. The SFDP26 has been applied to resident education [33] and has been translated and validated in additional languages [23, 34, 35]. For example, a teaching evaluation form, which was based on the SFDP26 and our institution’s Faculty Resident Electronic Evaluation System, was developed and tested among residents [20]. Research on clinical teaching of PA students found that characteristics of an effective and ineffective preceptor aligned with themes defined in the SFDP [36]. Two other studies used allied health students (including some PA students) to evaluate effective clinical instruction and found similar results [37, 38]. The PACRE instrument described in this study is unique in that it was specifically designed for and tested in a PA student population and focused on the overall rotation experience, not just clinical teaching.

The current study builds on prior work regarding student and rotation factors associated with perceived quality of the rotation. We found that rotation evaluation scores were correlated with student gender. Although we are unaware of studies exploring relationships between gender and rotation evaluation scores, previous work has demonstrated an association between medical student gender and medical school performance [39, 40]. We found that clinical rotation evaluation scores were associated with rotation specialty, feeling prepared for the rotation, and a better perception of the value of the rotation. Studies of medical students and residents have demonstrated that rotation quality is related to rotation specialty [23, 41], clinic schedule design [42], learning climate [43], requirements for overnight call [44], quality of feedback [44, 45], caseload [46], continuity [46], and level of faculty involvement [46]. In our study, associations between rotation quality and rotation specialty suggest that differences between specialties exist and that future studies could focus on elucidating these differences. The finding that rotation evaluation scores correlated with being prepared for the rotation is concordant with the current trend in medical schools to offer transition courses for early clinical experiences [47].

The PACRE questionnaire has compelling validity evidence. A common approach to validity in medical education research includes content, internal structure, and relations to other variables evidence [48]. Content validity for the PACRE questionnaire is based on published assessments of clinical teaching [12, 14, 15, 20–29], iterative revision of instrument items, and pilot testing. Internal structure validity is supported by a single factor to measure student perceptions’ of clinical rotations and excellent internal consistency reliability. Relations to other variables validity evidence is demonstrated by associations between clinical rotation evaluation scores and gender, rotation specialty, feeling prepared for the rotation, and viewing the rotation as valuable. Future study should determine if associations between PACRE questionnaire scores and other rotation outcomes including knowledge assessments exist.

### Limitations and strengths

First, all students did rotations through 2 programs, which could limit the generalizability of the findings. However, analyses of published medical education studies indicate that most are single institution studies [49]. Second, the majority of responders in this study were female, which may limit generalizability. Third, while the response rate in this study was excellent, there could be differences between those that did and did not complete the survey. Fourth, responses from the PACRE instrument are reaction outcomes rather than the higher outcomes of learning, behavior, or results [50]. Yet, reviews of medical education research suggest that reaction-level outcomes are commonly reported [49]. Fifth, the utilization of 124 surveys from 33 students represents a relatively small number for factor analysis. Sixth, the SFDP questionnaire was originally developed for assessing only preceptors and our utilization of the SFDP framework includes application of items to both preceptors and the program. However, most of the items reflect students’ perceptions of their preceptors, the items that are applied to the program are applied in ways that are true to the item’s original intent (e.g., “The rotation goals were stated clearly”), and we note that an advantage is that ours is the first study to provide robust validity evidence for use of the SFDP framework for evaluation of students perceptions of a PA program. Seventh, PACRE evaluation scores should be considered in the context of other outcomes including faculty evaluations and knowledge assessments (e.g. rotation examinations, observed structured clinical examinations) to fully evaluation a rotation. Finally, certain statistically significant score differences in this study (e.g., general practice PACRE score = 4.6 versus surgical rotations PACRE score = 4.1; *p* = 0.006) may seem small; nonetheless, in many education settings the inflation and range restriction of assessment scores is very narrow, and such magnitudes of difference could potentially separate the best rotations from the rest. Strengths of this study include a rigorous survey development process, use of a dedicated survey research program, robust statistical methods to establish instrument validity, and high response rate.

## Conclusions

This study has important implications for leaders of PA clinical rotation experiences. First, we believe that this is the first report of a valid method for assessing PA clinical rotation experiences. Application of the PACRE questionnaire could inform rotation directors about ways to improve clinical experiences. Given that the demand for PAs is rapidly increasing, the PACRE questionnaire could provide a consistent technique for ensuring that rotations provide meaningful teaching and clinical exposure. Second, the findings of this study suggest that PA students must be adequately prepared to have a successful experience on their rotations. PA programs should consider offering transition courses like those offered in many medical schools to prepare their students for clinical experiences. Third, variability exists among specialties regarding perceived quality of rotations. PA programs should work to provide standard requirements for rotations such as a curriculum, evaluation standards, competencies, and clinical exposure. Future research should explore whether additional rotation characteristics (e.g., didactic content, evaluation methods, call schedules) and educational outcomes (e.g., learning, behavior change) are associated with PACRE scores.

## Abbreviations

PA: Physician assistant
PACRE: PA clinical rotation evaluation
SFDP: Stanford Faculty Development Program
